# Conceptual Integration and Empirical Validation of a Unified Taxonomy: Quantitative Data Analysis for Virtual Learning Environments

**DOI:** 10.3389/fpsyg.2022.814592

**Published:** 2022-04-25

**Authors:** Melanie Moreno-Barahona, Blanca Fraijo-Sing, Ghozlane Fleury-Bahi, Oscar Navarro-Carrascal, Cesar Tapia-Fonllem

**Affiliations:** ^1^Programs of Master and Doctorate in Psychology, University of Sonora, Hermosillo, Mexico; ^2^Graduate Programs, Laboratoire de Psychologie des Pays de la Loire, University of Nantes, Nantes, France; ^3^Graduate Programs, Laboratoire UPR CHROME, Université de Nîmes, Nîmes, France

**Keywords:** educational affordances, taxonomic crossover, psychometrics, empirical validation, virtual learning environments (VLE)

## Abstract

Specific classes of cyberspaces emphasize different types of digital transactions given the user’s context, thus making it essential to take into account what these environments can afford. In this way, we can portray the niches of technological use as ecologies of particular possibilities and compare how they differ between distinct spheres of human life. The present research is focused on describing the conceptual integration of a taxonomic crossover between Virtual Learning Environments and Educational Affordances of Technology, while also performing empirical testing and determining the psychometric properties in a scale regarding the aforementioned taxonomy. The study sample consisted of 320 students in the departments of foreign languages from three different universities in Sonora (northwestern region of Mexico). Students were given a questionnaire of 21 items organized into four subscales with a Likert-type response option to measure the notions concerning their usage of Virtual Learning Environments. Internal consistency procedures and confirmatory factor analysis by means of Cronbach’s alpha and Structural modeling support the derived factorial structure, which contains Cyber-Communications, Virtual Behavior Settings, Virtual Communities, and Availability and Access to Connectivity. This structure traces the environmental properties perceived by learners in a virtual environment. Results sustain the initial conceptual construction regarding the proposed taxonomy, conclude that the ‘Virtual Learning Environments Questionnaire’ demonstrates adequate psychometric properties, and validate it as a fitting measure to assess the perceived psychological experience of students in a digital educational setting.

## Introduction

Categories and classifications are linked to everything humans do, ranging from the worlds where events happen to their complexities and the relationships between them. Categories as material or symbolic tools affect society in several ways: they are assigned, can become labels chosen for different events, and, in turn, can become statistical artifacts (Bowker and Leigh-Star, 2000). Without a classification, there would be no advanced conceptualization, reasoning, or data analysis. Classifications as taxonomies may refer to both the process and the result. Thus, the term taxonomy is reserved for a theoretical classification of empirical entities. Taxonomical methods, in general, begin with a set of observed data that are measured in a string of variables. Afterward, various techniques, which are traditionally quantitative, are used to group cases on their general resemblance (Bailey, 1994). The present research seeks to develop an exhaustive taxonomy and take it, with an empirical proof, to the operational or indicator level (Bailey, 1984, 1990) by an integration and junction of the concepts that shape each of the selected taxonomies. In this manner, we intend to characterize the main bodies of information and specify the methods that achieve this taxonomic cross.

Numerous concepts and constructs describing virtual learning environments, as well as their corresponding measures, have been proposed in the literature. Research on this topic has evolved to include several instruments designed to evaluate different learning environments by measuring variables such as the efficacy of learners and teachers that engage in them (Chard, 2006); Intrinsic Motivation in virtual learning environments (Fırat et al., 2017); the perceived quality of educational services provided by virtual learning environments (Martínez-Argüelles et al., 2013); scales developed to measure users’ engagement in specific virtual environments (Lee et al., 2019; Olivetti et al., 2020; Rojabi, 2020) and The perception of students about pedagogical models and standards in virtual learning environments (Barari et al., 2020; Torres Martín et al., 2021). While these contributions remain to be very significant to the field, they do not provide a general view on virtual learning environments as they focus on the environment’s isolated qualities. The taxonomy and corresponding scale presented here differ from prior research because it is sustained by both the educational affordances and spatial qualities of the components of cyberspace, therefore testing the integration of existing taxonomies. This integration will allow to capture the information more completely and to better evaluate virtual learning environments’ spatiotemporal qualities and the psychological experience of learners traversing them through their learning process.

The conceptual integration as proposed in this study is focused on describing different components of the cyberspaces or virtual environments, given their potential in learning processes. These components reference the capacity of the Internet and the cyberspace to bring sources of information closer to electronically simulated “virtual” places that are physically distant (Stokols and Montero, 2002). A virtual environment is defined as the experience of being surrounded by an environment synthesized by a computer, mobile device, or cyberspace, which might allow us to state that these types of environments move beyond a three-dimensional context, unlike physical spaces (Stokols, 2018a). In light of their ease of use on mobile devices, virtual environments allow for an interaction with the content and other users without regarding such a device as a computer, but rather as a space and an extension of their habitual daily practices (Lindaman and Nolan, 2015).

Therefore, we agree with the categorization of different components of cyberspace as conceived by Stokols (2018a), where distinct units of cyberspace emphasize different types of digital transactions. This categorization comprises Episodic Cyber Communications, that refer to conversations or exchanges in relatively short or designated time periods that are not as immersive as more extensive online interactions (e.g., E-mail, WhatsApp, Facebook Messenger, Instagram, Skype, and Zoom); Virtual Behavior Settings, meaning sites that stay online during longer periods of time and develop a symbolic sense of “space” or “place.” These are frequently built around particular goals or activities (e.g., Blogs or web pages of some particular theme, virtual libraries, different learning management systems such as Moodle and Schoology); and Virtual Communities, the most socially immersive kind of these cyberspaces, which makes reference to groups that involve recurring interactions between participants, who develop a shared identity and a virtual sense of camaraderie. Said cyberspaces often depict members of a community whose interactions and encounters are portrayed on an interface or screen (e.g., Classcraft, learning communities and support groups on Facebook, forums for questions, tutorials, and guides, or sites to make comments and receive feedback on a specific topic).

Other authors have mentioned that, by using taxonomies about virtual environments, we must, in turn, consider what these environments may afford us. Thusly, we may portray the niches of technological use as ecologies of particular possibilities and compare how they differ between specific contexts (Mitev and De Vaujany, 2013). On the topic of what technologies or digital environments afford us in an educational setting, we selected several categories from existing research that explore the *Educational Affordances of wearable technologies* and *Affordances of Information and Communication Technologies*. In this article, we have retrieved mutual components to conceive a category that integrates the affordances of Accessibility, Diversity, Communication, Presence, and Distribution (Boyle and Cook, 2004; Conole and Dyke, 2004; Bower and Sturman, 2015).

Finally, when contemplating cyberspace categories and what they afford us in regard to education, it is pertinent to comment on the material and physical possibility of the availability and access to the connectivity that students have in their places of study. This concept refers to the availability of equipment and services for connectivity in the learning environment, such as Hardware, Software, Internet connection (broadband, wireless, or mobile data), and Educational platforms that are used in everyday learning practices, since the access and usage of these resources in education improves quality, enhances creative thought, is associated to productivity and efficiency of educational results, and facilitates both the teaching and learning process (Siddiquah and Salim, 2017).

**FIGURE 1:**
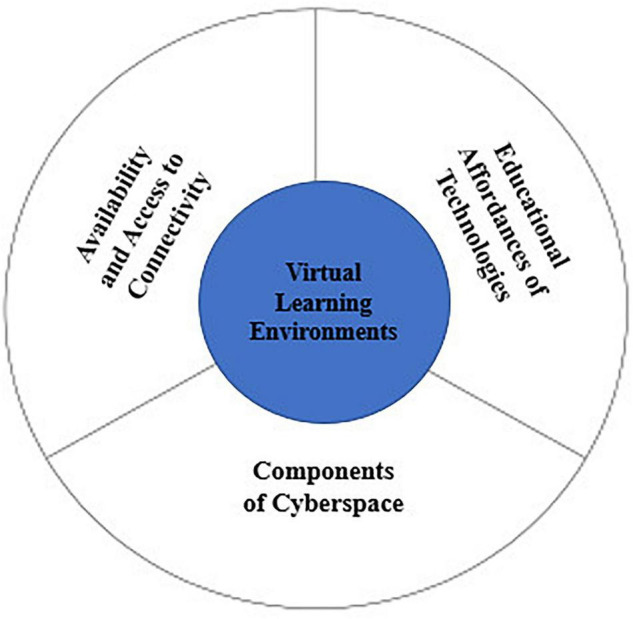
Integrated taxonomy for virtual learning environments.

The taxonomy comprised by the aforementioned variables may be illustrated in the following manner (see Figure 1).

## Methodology

### Participants

The study sample consisted of 320 students (Adults ≥18 years, *M* = 19.4) in the departments of foreign languages from Universidad del Valle de Mexico, Instituto Tecnológico de Hermosillo, and Universidad de Sonora. This sample included 115 male students and 205 female students, all of them located in the city of Hermosillo, Sonora in Northwestern Mexico. All participants were invited and then volunteered to partake in the online study. Participants also completed several other questionnaires related to language learning variables as part of a broader online study regarding language learning environments.

### Instrument and Measures

A questionnaire, called the Virtual Learning Environment Questionnaire (VLEQ), was based on the Taxonomy for Virtual Learning Environments, and developed specifically for its use in this study. It contained 21 items with a closed-ended Likert five-point scale response option (ranging from 0 – strongly disagree, to 5 – strongly agree). The VLEQ comprised four main sections: (1) Episodic Cyber Communications, (2) Virtual Behavior Settings, (3) Virtual Communities, and (4) Availability and Access to Connectivity. Regarding the first three scales, each one of them aimed to assess the suitability of the environmental quality perceived from each of these types of digital spaces dedicated to learning. As for the fourth scale, it was designed to gather information about the students’ availability of equipment and services for connectivity.

### Procedure

The VLEQ Questionnaire development procedure began in November 2020, based on a literature review and structuration of the taxonomic cross in this study. This was carried out with a review of major studies in the area, regarding virtual environments and the affordances of information technologies. Review evolved as ensued by peer feedback, followed by the technique proposed by Bailey (1994), where the planned selection and precise combination of a set of criteria with empirical referents served as a foundation for the taxonomy, and as a result obtaining the 21 Item pool which was to be proved through factor analysis, in order to identify the underlying relationships between measured variables.

The questionnaire was written in Spanish, and it was made available to students from March to April 2021. The recruitment process was tailored to ensure that the learners received the questionnaire via a link shared by their teachers, and then answered during their online classes. The VLEQ link provided also contained the informed consent clause which was signed by all participants.

### Data Analysis

The classic strategy is one of the most prevalent practices in social research, which first consists in specifying the concepts or constructs and then measuring empirical cases of them. This strategy alludes to a basic type of indicator classification called a “three-level measurement model,” where we find the concept, the corresponding empirical occurrence of the concept, and the indicator of both the concept and the empirical occurrence (Bailey, 1984). In these cases, typologies of a conceptual or empiric nature can only be abstracted through a measurement process which objectively identifies empirical cases for each conceptual category by measuring their correspondence (Bailey, 1994).

The main form of data analysis to be presented here is the results of the analysis from structural modeling procedures used to determine the taxonomy’s empirical validity and therefore the VLEQ’s reliability and construct validity. Specifically, we aimed to provide the first test of the factorial structure, presented here in two stages. The first step was to execute an Internal consistency analysis by means of Cronbach’s alpha reliability coefficient employing SPSS software. This coefficient was chosen in accordance with the needs of the study, given that it refers to the degree that the items of a measurement altogether reflect a simple latent variable (Cohen et al., 2012). Secondly, we demonstrated the validity of the approach by performing a confirmatory factor analysis by the means of structural equation modeling and a covariance analysis between the four factors developed with the proposed taxonomy. The modeling of latent variables provides convergent and discriminative evidence of the validity of the construct (Cohen and Swerdlick, 2006); the latter is assessed by examining the chi-square (χ^2^) statistic and its degrees of freedom. Moreover, other indexes used to estimate model fit include the Normed Fit Index (NFI), Non-normed Fit Index (NNFI), Comparative Fit Index (CFI), and Root-Mean-Square Error Approximation (RMSEA); all of which can be computed in EQS software.

## Results

### Reliability and Internal Consistency

All scales in this study were individually tested. Since, for the most part, tests are not always assumed to be homogeneous; but rather, it is sought that each of its scales, separately, measures a set of traits or characteristics different from those measured by the other scales included in the test (Cohen and Swerdlick, 2006). Table 1 shows the internal consistency of the scales used in the study. We can observe that all round, scale coefficients are both statistically significant and strongly correlated (see Table 2), presenting high Cronbach’s alpha values (α ≥ 0.75), hence, demonstrating an excellent internal consistency and reliability coefficient of the questionnaire.

**TABLE 1:** Internal consistency of the scales.

Scale	Cronbach’s alpha (α)
**Virtual Learning Environment Questionnaire**	
Episodic Cyber Communications	0.94
Virtual Communities	0.91
Virtual Behavior Settings	0.93
Availability and Access to Connectivity	0.91

**TABLE 2:** Pearson’s correlation coefficients between the scales contained in the Virtual Learning Environments Questionnaire.

	CC	VBS	Vir. C	AAC
CC	1			
VBS	0.789**	1		
Vir. C	0.702**	0.800**	1	
AAC	0.582**	0.648**	0.617**	1

**p < 0.01; n = 320.

*CC, Cyber Communications; VBS, Virtual Behavior Settings; Vir. C, Virtual Communities; AAC, Availability and Access to Connectivity.*

### Confirmatory Factor Analysis and Model Fit

**FIGURE 2:**
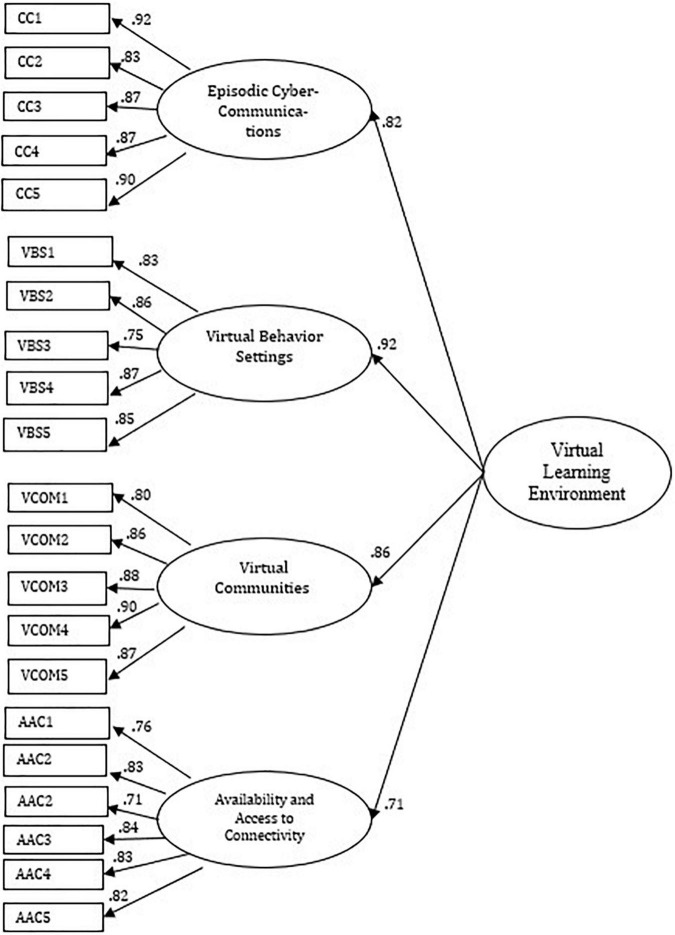
Structural equation model of second-order factor virtual learning environment with four first-order factors. All factor loadings are significant (*p* < 0.05). Values of errors are not reported. Goodness of fit: χ^2^ = 668.04 (*df* = 179), *p* = 0.000; BBNFI = 0.92, BBNNFI = 0.93, CFI = 0.94; RMSEA = 0.08. Circles indicate latent variables, and boxes indicate the number of the item.

With regards to model fit and its interpretation, Figure 2 shows the obtained model for the Virtual Learning Environments Taxonomy, which shows a second-order factor explained by four first-order factors. We employed absolute fit indexes such as chi-square statistic to assess the degree to which the proposed structure and the actual data variance compare (Bentler, 1995). By observing it, we can corroborate that the indicators of goodness of statistical adjustment were in this case significant (χ^2^ = 668.04, *df* = 179, *p* = 0.000). However, we may also remark that all factorial loads are equal to or greater than 0.70, and adjunct or practical goodness of fit indexes (NFI = 0.92, NNFI = 0.93, CFI = 0.94, RMSEA = 0.08) show that the model is well supported by the amount of observed data contained in our sample since fit index all values are equal to or greater than 0.90 and RMSEA ≤ 0.08 (Bentler, 1990; Corral-Verdugo, 1995; Ullman and Bentler, 2012), thus, proving an adequate factorial structure and model fit regarding its practical indicators, which brought additional information about the value of the hypothesized model (Thompson, 2004; Shi et al., 2019; Park and Kim, 2021).

## Discussion

The results from this study point to the conclusion that the conceptual integration and empirical validation of the unified taxonomy for Virtual Learning Environments were solidly constructed and verified. Once indicators are classified into their designated groups, discriminant analysis works with all variables linearly to make a prediction as to the group to which the indicator belongs. This means that a collection of empirical cases assigned to groups and a set of continuously measured variables come to represent the conceptual-indicator-empirical structure obtained via discriminant analysis (Bailey, 1984).

Therefore, by acknowledging this model, this research is a first step toward a more profound understanding of virtual learning environments and study of the scope and complexity of the cybersphere in educational settings. Literature on virtual or digital environments indicates we should consider the cybersphere as a broad domain of environmental influence and search for ways to assess the varied outcomes of virtual life in relation to people’s contexts (Stokols, 2019a). Here, we explored some of the contextual relationships embedded in virtual learning environments, approaching the study of how technologies and virtual settings may afford a sense of ecological presence (Frielick, 2004). While touching on the impact of virtual environments in educational settings, we can also make an emphasis on how digital communications and the components of the cybersphere have an influence on a person’s day-to-day activities (Stokols, 2019b). In this case, an influence on students’ activities and the qualities they perceive from their digital environments. Moreover, the model in this study may shed a light on strategies for digitalized or remote learning and teaching, tactics for adapting to change within the transition to online learning, and the design of digital learning spaces (Abdelhafez, 2021; Lyu, 2021).

A key strength of this research lies within the fact that the newly proposed questionnaire (VLEQ) specializes in studying some of the perceived affordances in virtual learning environments solely based on the items derived from a taxonomic cross which included theoretical categories that had not been tested with empirical cases before. These indicators were subjected to empirical testing procedures in order to obtain and analyze the psychometric properties which determined the validity of the taxonomy portrayed in the questionnaire.

Even in light of the results obtained, a significant limitation in this study relates to generalization, since we worked with data from only 320 second language learning students at university level, therefore the results cannot be claimed to universally be the case for all learners and educational degrees. Furthermore, this work only offers limited aspects regarding each category for virtual learning environments, future directions may point to the improvement of several features of the taxonomy. These may be explored by further development of the questionnaire, such as adding more examples to better describe the different kinds of cyberspaces or including more items in order to describe other affordances of information technologies.

The obtained results justify further development of the method, several interesting aspects may be explored to a greater extent by adapting the instruments and methods of this study to the needs of other populations in different educational levels. Future works should include different kinds of students (including different areas and fields of knowledge), while also associating their virtual, physical, and social contexts in order to further detail the interconnection between these environments and its impact on variables related to learning processes.

## Data Availability Statement

The original contributions presented in the study are included in the article/Supplementary Material, further inquiries can be directed to the corresponding author.

## Ethics Statement

The studies involving human participants were reviewed and approved by the Committee of Ethics in Research (Comité de Ética en Investigación) of the University of Sonora. The patients/participants provided their written informed consent to participate in this study.

## Author Contributions

MM-B contributed to the conceptualization and design of the study, acquired the data, ran formal analysis, and organized the databases. BF-S and GF-B contributed to supervising the study, methodological tasks, and data interpretation. CT-F made substantial contributions by editing and revising the manuscript critically for important intellectual content. MM-B and ON-C provided the writing of the original draft. All authors contributed to manuscript revision and read and approved the submitted version.

## Conflict of Interest

The authors declare that the research was conducted in the absence of any commercial or financial relationships that could be construed as a potential conflict of interest.

## Publisher’s Note

All claims expressed in this article are solely those of the authors and do not necessarily represent those of their affiliated organizations, or those of the publisher, the editors and the reviewers. Any product that may be evaluated in this article, or claim that may be made by its manufacturer, is not guaranteed or endorsed by the publisher.
