# Supplementary material for: Conceptual Integration and Empirical Validation of a Unified Taxonomy: Quantitative Data Analysis for Virtual Learning Environments
Source: Front Psychol. 2022 Apr 25;13:814592. doi: 10.3389/fpsyg.2022.814592 (PMC9084617; doi:10.3389/fpsyg.2022.814592)
Supplement: Supplementary file 1 [file Data_Sheet_1.PDF]

Supplementary Material

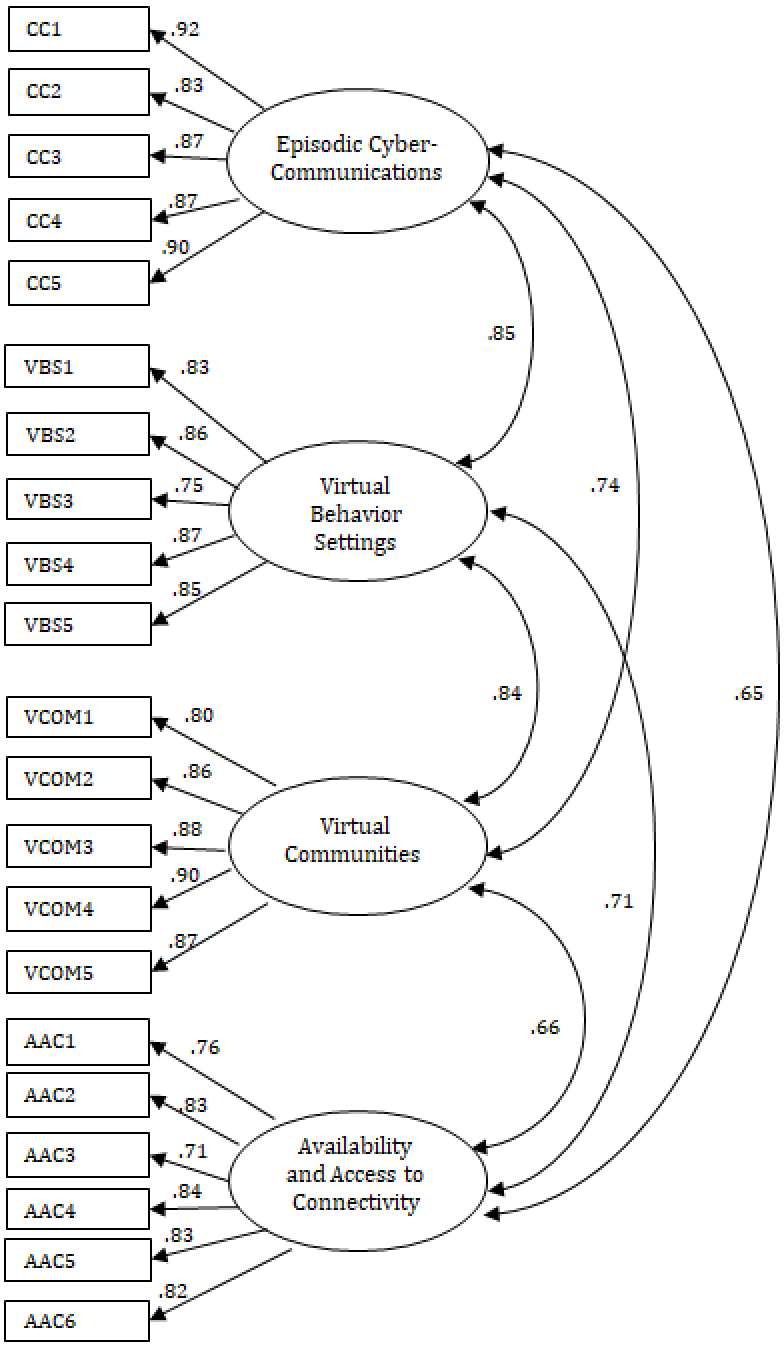

Supplementary Figure 1. *Confirmatory factor analysis of Virtual Learning Environments. All factor loadings and covariances between factors are significant (p <.05). Values of errors are not reported. Goodness of fit:* $\chi 2$ *= 507.10 (176 df), p= .000; NFI=.92, NNFI = .93, CFI = .95; RMSEA=.07.*

Virtual Learning Environments Questionnaire

Please indicate the extent to which you agree with the statements listed here. Consider each of the statements in relation to the subject or content you are currently learning in this class. It is important not to guess, but to give a realistic estimate of your opinion. Please mark only one number between 0 and 4 for each statement. Thanks!

Episodic cyber communications Scale

This section refers to the use of e-mail, text messages such as **E-mail, WhatsApp, Facebook Messenger, Instagram, Videoconferences like Skype or Zoom** in your daily learning practices (whether at home, school or anywhere else).

Items	*Min*	*Max*	$\bar{x}$	σ
I use cyber communications because I think they are efficient (e.g., I can communicate with a classmate quickly and without complications).	0	4	3.48	1.038
I make use of cyber communications because I think they provide a wide range of different experiences for my learning.	0	4	3.19	1.090
I use cyber communications because I think they are a way to be active in interaction with others.	0	4	3.26	1.088
I use cyber communications because I think they are useful for the distribution and acquisition of resources/materials for learning (e.g., sharing my notes or class notes).	0	4	3.30	1.056
I use cyber communications because I think they help me get in touch with others in the place or place where I am from my usual device.	0	4	3.44	1.072

Supplementary Table 1. Descriptive statistics for the Episodic cyber communications Scale. n = 320; $\bar{x}$ = mean; σ= standard deviation.

Virtual Communities Scale

This section refers to the use of e-mail, text messages such as **Classcraft, learning communities and support groups on Facebook, forums for questions, tutorials, and guides, or sites to make comments and receive feedback on a specific topic** in your daily learning practices (whether at home, school or anywhere else).

Items	***Min***	***Max***	$\bar{x}$	σ
Virtual communities provide information that is well organized and easy to find.	0	4	2.80	1.045
I reach out to virtual communities because they offer variability of learning experiences (e.g., consult fan pages / forums to learn more about a topic, read user reviews).	0	4	2.88	1.121
I reach out to virtual communities because they are a way of being present and active in a community or group from which I can learn.	0	4	2.82	1.195
I reach out to virtual communities because in them it is easy to create, find and share with others the materials that could contribute to our learning.	0	4	2.98	1.158
Virtual communities can provide me with information at the time it is required from my usual device (e.g., ask a question or query in a group while I am heading home on the bus).	0	4	3.13	1.085

Supplementary Table 2. Descriptive statistics for the Virtual Communities Scale.
n = 320; $\bar{x}$ = mean; σ= standard deviation.

Virtual Behavior Settings Scale

This section refers to the use of e-mail, text messages such as **Blogs or web pages of some particular theme, virtual libraries, different learning management systems such as Moodle and Schoology** in your daily learning practices (whether at home, school or anywhere else).

Items	***Min***	***Max***	$\bar{x}$	σ
In these virtual spaces, obtaining information is fast and efficient.	0	4	3.14	1.018
I go to these virtual spaces because they are diverse and varied (e.g., I can find different ways to learn and practice such as visiting a blog or a web page).	0	4	3.21	1.062
I frequent these virtual spaces because they give a sense of presence when interacting with other people.	0	4	2.87	1.133
I go to these virtual spaces because through them you can create, acquire and distribute materials for my learning (e.g., Share with classmates a book that you download in a digital library).	0	4	3.25	1.046
In these virtual spaces, information is accessible at any time and place from my usual device (e.g., search for information on a specialized web page from wherever I am).	0	4	3.36	1.019

Supplementary Table 3. Descriptive statistics for the Virtual Behavior Settings Scale. n = 320; $\bar{x}$ = mean; σ= standard deviation.

Availability and Access to Connectivity Scale

This section refers to the availability of equipment and services for connectivity on your campus (**Hardware, Software or programs, Internet connection, etc.**) that are used in daily learning practices.

Items	***Min***	***Max***	$\bar{x}$	σ
I have sufficient resources and efficient means for connectivity (Internet access) inside and outside my school.	0	4	2.83	1.313
In my school/university there are virtual schooling services or platforms that allow students to expand their study opportunities.	0	4	3.20	1.106
My school/university has a training program/course/guidance subject on technology management and virtual learning platforms.	0	4	2.90	1.251
My school/university provides access to computers and other equipment that give students the tools to work on their academic assignments.	0	4	3.11	1.178
My school/university provides access to an information base (databases, repositories, digital libraries, etc.) that is constantly expanding online.	0	4	2.91	1.373
My school/university provides access to a variety of free software that allows students work on different academic tasks and assignments.	0	4	2.95	1.330

Supplementary Table 4. Descriptive statistics for the Availability and Access to Connectivity Scale. n = 320; $\bar{x}$ = mean; σ= standard deviation.

Original Item Pool for the Virtual Learning Environments Questionnaire (Spanish Version)

Items

Escala de Ciber comunicaciones episódicas

1- Utilizo las ciber comunicaciones porque pienso que son eficientes (ej. Puedo comunicarme con un compañero de clase de manera rápida y sin complicaciones).
2- Hago uso de las ciber comunicaciones porque pienso que brindan una amplia gama de experiencias diferentes para mi aprendizaje.
3- Utilizo las ciber comunicaciones porque pienso que son una manera de estar activo en la interacción con otros.
4- Empleo las ciber comunicaciones porque pienso que resultan útiles para la distribución y adquisición de recursos/materiales para el aprendizaje (ej. Compartir mis notas o apuntes de clase).
5- Utilizo las ciber comunicaciones porque pienso que ayudan a ponerme en contacto con otros en el lugar o sitio donde me encuentre desde mi dispositivo habitual.

Escala de Comunidades Virtuales

6- Acudo a comunidades virtuales porque ofrecen variabilidad de experiencias de aprendizaje (ej. consultar páginas de "fans" /foros para saber más sobre un tema, leer críticas de usuarios).
7- Acudo a comunidades virtuales porque ofrecen variabilidad de experiencias de aprendizaje (ej. consultar páginas de "fans" /foros para saber más sobre un tema, leer críticas de usuarios).
8- Acudo a comunidades virtuales porque son una manera de estar presente y activo/a en una comunidad o grupo del que puedo aprender.
9- Recurro a comunidades virtuales porque en ellas es sencillo crear, encontrar y compartir con otros los materiales que podrían contribuir a nuestro aprendizaje.
10- Las comunidades virtuales me pueden proporcionar información en el momento que se requiera desde mi dispositivo habitual (ej. Realizar una pregunta o consulta en un grupo mientras me dirijo a casa en el autobús).

Escala de Escenarios de Conducta Virtuales

11- En estos espacios virtuales la obtención de información es rápida y eficaz.
12- Recurro a estos espacios virtuales porque son diversos y variados (ej. Puedo encontrar diferentes formas de aprender y practicar como visitando un blog o una página web).
13- Frecuento estos espacios virtuales porque dan un sentido de presencia al interactuar con otras personas.
14- Recurro a estos espacios virtuales porque a través de ellos se puede crear, adquirir y distribuir materiales para mi aprendizaje (ej. Compartir con compañeros un libro que descargue en una biblioteca digital).
15- En estos espacios virtuales la información es accesible en cualquier momento y lugar desde mi dispositivo habitual (ej. Buscar información en una página web especializada desde donde me encuentre).

Escala de Disponibilidad y Acceso a la Conectividad

16- Cuento con recursos suficientes y medios eficientes para la conectividad (acceso a internet) dentro y fuera de mi escuela.

17- En mi escuela/universidad se dispone de servicios o plataformas de escolarización virtual que permiten ampliar las oportunidades de estudio de los alumnos.

18- Mi escuela/universidad cuenta con un programa de capacitación/curso/materia de orientación sobre manejo de tecnologías y plataformas virtuales de aprendizaje.

19- Mi escuela/universidad facilita el acceso a computadoras y otros equipos que brindan a los estudiantes las herramientas para trabajar en sus deberes académicos.

20- Mi escuela/universidad brinda acceso a una base de información (bases de datos, repositorios, bibliotecas digitales, etc.) que se encuentra en constante expansión a través de Internet.

21- Mi escuela/universidad proporciona acceso a una variedad de software gratuito que permite a los estudiantes realizar diferentes tipos de trabajos.

Supplementary Table 5. List of the original items contained in the Spanish version of the Virtual Learning Environments Questionnaire.
